# Comparative Genomics of the World's Smallest Mammals Reveals Links to Echolocation, Metabolism, and Body Size Plasticity

**DOI:** 10.1093/gbe/evae225

**Published:** 2024-10-21

**Authors:** Marie-Laurence Cossette, Donald T Stewart, Aaron B A Shafer

**Affiliations:** Department of Environmental Life Sciences Graduate Program, Trent University, Peterborough, ON, Canada; Department of Biology, Acadia University, Wolfville, NS, Canada; Department of Environmental Life Sciences Graduate Program, Trent University, Peterborough, ON, Canada; Department of Forensic Science, Trent University, Peterborough, ON, Canada

**Keywords:** comparative genomics, genome assembly, accelerated regions, positive selection, gene duplication

## Abstract

Originating 30 million years ago, shrews (Soricidae) have diversified into around 400 species worldwide. Shrews display a wide array of adaptations, with some species having developed distinctive traits such as echolocation, underwater diving, and venomous saliva. Accordingly, these tiny insectivores are ideal to study the genomic mechanisms of evolution and adaptation. We conducted a comparative genomic analysis of four shrew species and 16 other mammals to identify genomic variations unique to shrews. Using two existing shrew genomes and two de novo assemblies for the maritime (*Sorex maritimensis*) and smoky (*Sorex fumeus*) shrews, we identified mutations in conserved regions of the genomes, also known as accelerated regions, gene families that underwent significant expansion, and positively selected genes. Our analyses unveiled shrew-specific genomic variants in genes associated with the nervous, metabolic, and auditory systems, which can be linked to unique traits in shrews. Notably, genes suggested to be under convergent evolution in echolocating mammals exhibited accelerated regions in shrews, and pathways linked to putative body size plasticity were detected. These findings provide insight into the evolutionary mechanisms shaping shrew species, shedding light on their adaptation and divergence over time.

**Significance**

Shrews belong to one of the largest mammalian families and are widespread across the globe. Many shrew species have developed unique traits, yet the genetic basis of these adaptations remains poorly understood. In our study, we performed comparative genomic analyses to explore genomic differences in shrews compared to other mammals. We discovered that different genomic regions between shrews and other mammals were linked to echolocation, nervous system development, and metabolic processes. This research provides a list of candidate genes for further investigation, offering new insights into the genetic basis of unique phenotypes in shrews.

## Introduction

Mammals have undergone successive episodes of rapid diversification resulting in the emergence of over 6,000 extant species worldwide (Burgin et al. 2018). Shrews (Soricidae) appeared over 30 million years ago (Churchfield 1990), and have become one of the most diverse mammalian taxa, having undergone numerous colonization, speciation, and extinction events (Reumer 1989). Shrews consist of more than 400 extant species (Burgin et al. 2018) classified into three sub-families: Soricinae (red-toothed shrews), Crocidurinae (white-toothed shrews), and Myosoricinae (African shrews) (Hutterer 2005). This diversification is also reflected in the diverse karyotypes both between (Schlitter et al. 1999) and within species (Wójcik et al. 2002; White et al. 2019), including sex chromosomes (Sharman 1956).

Shrews have successfully colonized a diverse array of habitats across the globe (George 1986, 1988) ranging from tropical forests to grasslands and arid regions (Churchfield 1990). This has led to a range of adaptations, such as echolocation to navigate their surroundings (Tomasi 1979; Forsman and Malmquist 1988; Chai et al. 2020), aquatic diving capabilities to hunt prey (Mendes-Soares and Rychlik 2009), venomous saliva for predation (Kita et al. 2004), and metabolic shifts (Thomas et al. 2023) and reversible body size changes to survive winter (Lázaro and Dechmann 2021). Shrews are among the smallest, shortest-lived mammals (Churchfield 1990) and have an extremely high metabolic rate (Ochocińska and Taylor 2005) which requires them to consume up to 125% of their body weight in food each day (Churchfield 1990). These adaptations and overall diversity of shrews make for a unique system to investigate genome evolution in the context of mammalian diversity and evolution.

Approximately 10% of the human genome appears evolutionary constrained across mammals (Christmas et al. 2023). These highly conserved regions are predicted to be functionally important (Ponting 2017; Bi et al. 2023), but may occasionally exhibit lineage-specific increases in nucleotide substitutions, which are referred to as accelerated regions (ARs) (Pollard et al. 2006a; Ferris et al. 2018). ARs are thought to contribute to species-specific traits (Ferris et al. 2018; Levchenko et al. 2018) and can result from evolutionary forces such as positive selection and GC-biased gene conversion (Pollard et al. 2006a; Hubisz and Pollard 2014; Ferris et al. 2018; Bi et al. 2023). Ferris et al. (2018) and Tollis et al. (2021) identified ARs enriched near immune system and DNA damage response genes in elephants (*Loxodonta*), which are known to show resistance to cancer. Similarly, human ARs have been associated with proteins hypothesized to be important in neurodevelopment (Pollard et al. 2006b).

On a larger scale, gene duplication provides new genetic material that can generate novel phenotypes that selection can act upon (Magadum et al. 2013). In bats (*Myotis*), repeated duplications of the protein kinase R (*PKR*) gene have been linked to immunity to viruses (Jacquet et al. 2022). Similarly, gene loss also has the potential to lead to phenotypic evolution and diversity (Sharma et al. 2018; Helsen et al. 2020). The loss of AMP deaminase 3 (*AMPD3*) in sperm whales (*Physeter macrocephalus*) has likely improved O_2_ transport (Sharma et al. 2018). Other variants, such as larger structural rearrangements, also contribute to genomic diversity and evolution (Damas et al. 2021, 2022), highlighting the complex nature of the genomic architecture underlying phenotypic evolution.

Comparative genomics can be used to identify unique and shared genomic features, providing insight on the genetic basis of diversity and patterns involved in species-specific traits. Understanding gene family evolution, including adaptive expansion (i.e. duplication of genes) and adaptive contraction (loss of genes), has increasingly become a focus of molecular evolutionary genetics as the number of fully sequenced genomes has been increasing (Rhie et al. 2021). Here, we assembled two de novo shrew genomes for the maritime shrew (*Sorex maritimensis*), which is endemic to Canada (Stewart et al. 2002), and the smoky shrew (*Sorex fumeus*) (supplementary fig. S1a, Supplementary Material online). Although restricted to Canada, the maritime shrew is part of the *Sorex* subgenus of *Sorex* shrews that are predominantly found in Eurasia (Fumagalli et al. 1999). The smoky shrew is a member of the subgenus *Otisorex*, members of which are predominantly found in the Nearctic region (George 1988). We included two previously assembled shrew genomes for the Etruscan shrew (*Suncus etruscus*) and the Eurasian common shrew (*Sorex araneus*) and compared them against 16 other mammal genomes (supplementary fig. S1b, Supplementary Material online). We aimed to uncover shrew-specific genomic changes associated with their distinctive phenotypes, with the prediction that shared genomic variants among shrew species will be associated with unique phenotypes and traits shared among these species. We focused on characterizing ARs, gene family duplications, expansion, and contraction events, and positively selected genes in shrew to provide insights into the evolutionary mechanisms driving shrew diversity and adaptation.

## Results

### Genome Assembly and Annotations

For the smoky shrew assembly (GenBank assembly accession: GCA_029834395.2), the five HiFi cells produced 115,806,042,303 bp of HiFi data, or ∼43× coverage from the estimated 2.7 GB genome (Cossette et al. 2023). The mean length of the HiFi reads was 14,652 bp. We generated 368,278,690 paired Hi-C reads. The final smoky shrew assembly was 2.87 GB with an N50 of 42.40 Mb and over 99% of the genome being in scaffolds >50 kb long (Table 1). Approximately 51.9% of the genome was made of repetitive sequences (Table 2). The BUSCO assessment was 93.4% complete, of which 91.8% were single-copy. Distribution plots showed a high-percentage of single-copy k-mers in the final assembly. We generated 890,398,361 raw 150 bp paired-end reads (∼110× coverage) and 96,242,024,700 bp of 10× reads. The maritime shrew genome was 2.43 GB (GenBank assembly accession: GCA_030324115.1) and comprised of 91,327 scaffolds with an N50 of 84.5 kb. A total of 69.8% of the assembly was in scaffolds over 50 kb long, and 41.7% of the genome was classified as repetitive sequences. Out of the BUSCO orthologs from the mammalian data set, 72.9% were complete with 70.6% being single-copy. The potential impact of this lower quality genome assembly on subsequent analyses is discussed below (see Discussion).

**Table 1:** Genome assembly statistics for the smoky shrew genome generated from PacBio HiFi long-reads and Hi-C reads, the maritime shrew genome generated from 10X long-reads and short-reads, and the common shrew and Etruscan shrew genomes downloaded from NCBI

	Smoky shrew	Maritime shrew	Common shrew	Etruscan shrew
Main genome assembly size	2.87 GB	2.43 GB	2.42 GB	2.47 GB
Main genome number of scaffolds	499	91,327	12,478	147
Main genome scaffold N/L50	42.4 Mb/23	84.5 kb/7,135	22.8 Mb/36	131.9 Mb/8
Main genome scaffold N/L90	17.4 Mb/49	15.3 kb/29,605	4.2 Mb/129	87.5 Mb/17
Max scaffold length	91.7 Mb	2.5 Mb	60.2 Mb	208.2 Mb
Number of scaffolds > 50 kb	365	14,697	473	70
% main genome in scaffolds > 50 kb	99.9%	69.8%	98.4%	99.9%
Genome coverage	43.0×	76.5×	120.0×	142.6×
Number of protein-coding genes	20,380	21,191	19,080	19,819

**Table 2:** Summary of repeats in the smoky and maritime shrew genomes in length occupied (bp) and percentage of the genome

	Smoky shrew	Maritime shrew
SINE	182,692,788 (6.36%)	141,858,027 (6.90%)
LINE	614,478,406 (21.38%)	366,060,918 (17.81%)
LTR elements	41,000,372 (1.43%)	30,015,478 (1.46%)
DNA transposons	10,884,988 (0.38%)	6,592,309 (0.32%)
Unclassified	500,269,535 (17.41%)	198,021,546 (9.63%)
Rolling-circles	686,527,20 (2.39%)	58,004,316 (2.82%)
Small RNA	20,490,319 (0.71%)	18,114,174 (0.88%)
Satellites	2,682,795 (0.09%)	2,057,146 (0.10%)
Simple repeats	39,822,407 (1.39%)	29,813,949 (1.45%)
Low complexity	9,066,276 (0.32%)	5,801,032 (0.28%)
Total	1,421,387,886 (51.86%)	856,338,895 (41.65%)

A total of 20,380 protein-coding genes were identified in the smoky shrew genome and 21,191 in the maritime shrew genome. The assembled maritime and smoky shrew mitochondrial genomes were 16,979 bp and 17,051 bp long, respectively; all 37 mitochondrial genes were present in both assemblies.

### Accelerated Regions

**Fig. 1.**
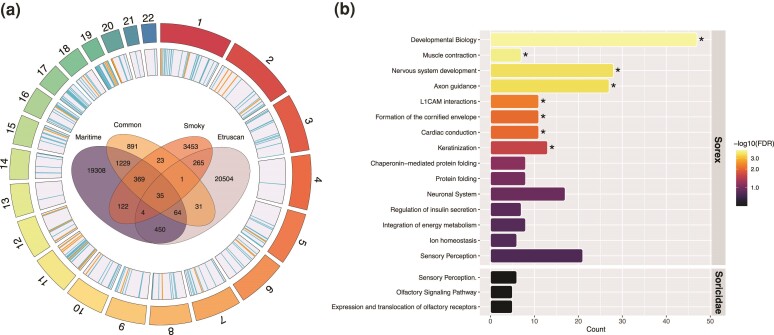
Accelerated region (AR) analysis results. (a) Location of ARs shared between *Sorex* species (*n* = 404) in blue and Soricidae (*n* = 35) in yellow mapped to the human (hg38) genome. Venn diagram for the number of overlapping ARs between shrew species. (b) Top Reactome pathways for overlapping ARs in *Sorex* and Soricidae. Asterisk represents significant pathways (5% FDR). Top pathways for individual shrew species can be found in the Supplemental (see supplementary fig. S2, Supplementary material online).

We identified 598,180 conserved regions (~1% of the genome) (supplementary table S1, Supplementary Material online) in our alignments, comparable to other mammalian studies (Ferris et al. 2018; Tollis et al. 2021). From these regions, 307,989, ∼51% of conserved regions, were in exons. We found 2,643 common shrew ARs in 1,627 genes, 21,581 maritime shrew ARs in 4,528 genes, 4,272 smoky shrew ARs in 2,257 genes, and 21,354 Etruscan shrew ARs in 6,787 genes (Table 3, supplementary table S1, Supplementary Material online). Soricidae shared 35 ARs, and the *Sorex* species shared 404 ARs (Fig. 1a). Notable ARs were found in genes related to the nervous system such as the growth associated protein 43 (*GAP43*), fibroblast growth factor receptor 1 (*FGFR1*), and class III β-tubulin (*TUBB3*) (Table 4). Also, genes involved in metabolic pathways such as the adiponectin receptor 1 (*ADIPOR1*) and glyceraldehyde-3-phosphate dehydrogenase (*GAPDH*) had shared ARs in *Sorex* and Soricidae, respectively (Table 4). The common shrew and maritime shrew genomes shared ARs in the cadherin related 23 (*CDH23*) and otoferlin (*OTOF*) genes, which are associated with echolocation capabilities in mammals (Shen et al. 2012; Table 4). The pathway analyses revealed genes associated with nervous system development, olfactory receptors, insulin and energy metabolism pathways, muscle contraction, and cardiac conduction as well as keratinization (Fig. 1b, supplementary fig. S2, Supplementary Material online).

**Table 3:** Summary statistics from each analysis per shrew species/node

	Smoky shrew	Maritime shrew	Common shrew	Etruscan shrew	*Sorex*	Soricidae
# accelerated regions	4,272	21,581	2,643	21,354	404	35
# gene duplications	2,328	2,658	2,722	2,306	62	42
# significant+/−orthogroups	+85/−8	+54/−503	+407/−1	+128/−36	+62/−5	+52/−18
# genes under selection	31	97	9	116	62	47

**Table 4:** Genes associated to the nervous system, auditory system, and metabolism in shrew species/nodes identified in each analysis based on the human hg38 annotations (NCBI accession: GCA_000001405.15)

	Node/species	Gene	Function
Accelerated regions	*Sorex sorex*	CDH23	Critical component of hair bundle formation (Palma et al. 2001)
	*Sorex sorex*	OTOF	Involved in vesicle release at the synapse between inner hair cells and auditory nerve fibers (Roux et al. 2006)
	*Sorex*	GAP43	Associated to neurite outgrowth and synaptic plasticity (Aigner et al. 1995; Lee et al. 2023)
	*Sorex*	TUBB3	Involved in axon regeneration (Latremoliere et al. 2018)
	*Sorex*	CPNE6	Regulates dendritic spine structural plasticity, learning and memory (Reinhard et al. 2016)
	*Sorex*	FGFR1	Necessary for hippocampal growth (Ohkubo et al. 2004)
	*Sorex*	ADIPOR1	Plays a role in glucose and lipid metabolism (Yamauchi et al. 2007)
	Soricidae	GAPDH	Involved in glycolysis pathway (Seidler 2013)
	Soricidae	CSNK2A1	Mutations associated with neurodevelopmental abnormalities (Okur et al. 2016)
Duplication/CAFE	*Sorex*	SRR	Involved in synaptic plasticity (Wong et al. 2020) and affects adult neurogenesis (Roychaudhuri et al. 2023)
	*Sorex*	PURA	Important for postnatal brain development (Khalili et al. 2003)
	*Sorex*	UGT8	Involved in the synthesis of glycosphingolipid of myelin (Dziȩgiel et al. 2010)
dN/dS	Soricidae *Sorex* *Sorex sorex* *S. fumeus* *S. maritimensis* *S. etruscus*	ANK2	Variants associated to cardiac and neurological disorders (York et al. 2022)
	*Sorex* *Sorex sorex* *S. maritimensis* *S. etruscus*	ANO10	Variants are known to cause spinocerebellar ataxia, a neurological disorder (Chrysanthou et al. 2023)
	Soricidae *Sorex* *S. maritimensis* *S. etruscus*	MYO9B	Modulates dendritic morphogenesis in the brain (Long et al. 2013)
	*S. fumeus* *S. etruscus*	RTN4R	Plays an essential role in regulating axonal regeneration and plasticity in the central nervous system (Kimura et al. 2017)
	Soricidae *S. maritimensis*	ACLY	Directs glucose metabolic fluxes to de novo lipogenesis (Li et al. 2023)
	*Sorex*	CH25H	Modulates cholesterol homeostasis (Zhao et al. 2020)

### Gene Family Size Evolution

**Fig. 2.**
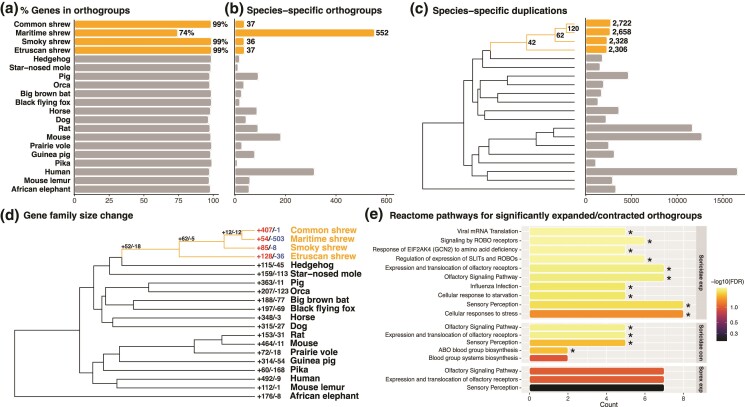
OrthoFinder and CAFE analyses results. (a) Percentage of genes from each species assigned to orthogroups. (b) Number of species-specific orthogroups. (c) Number of species-specific duplication events. Number of duplication events shared by all descendants of the shrew nodes are also represented. (d) Number of significantly (*P*-value < 0.01) expanded/contracted orthogroups across 20 mammalian species. Shrew nodes and branches are highlighted. (e) Top Reactome pathways for the significantly expanded/contracted orthogroups shared between Sorex and Soricidae species. Asterisk represents significant pathways (5% FDR).

Orthofinder assigned 476,077 (97.0% of total) genes to 20,489 orthogroups. Of these, 7,954 orthogroups included at least one ortholog from each of the 20 species and 1,810 orthogroups were species-specific. A total of 533 genes were identified as single-copy genes across all 20 mammals. The maritime shrew had 15,755 (74% of genes) genes assigned to orthogroups (Fig. 2a) and had the most species-specific orthogroups (Fig. 2b), which may be due to inaccurate gene predictions resulting from the fragmented assembly (Table 1). The three other shrew species’ assemblies had on average 37 unique gene families each (Fig. 2b). Orthofinder further identified 2,722 gene duplication events in the common shrew, 2,658 in the maritime shrew, 2,328 in the smoky shrew, and 2,306 in the Etruscan shrew (Fig. 2c, Table 3). There were 42 gene duplication events shared between all shrew species and 62 between the *Sorex* genus (Fig. 2c, Table 3).

The number of gene families undergoing expansion ranged between 904 and 1,476 in shrews with up to 407 significantly expanding gene families in the common shrew (Fig. 2d). The maritime shrew appeared to have undergone more contractions (*n* = 7,285) than any other shrew species which ranged between 629 and 1,598 contractions. This discrepancy may be due to incomplete gene predictions resulting from the fragmented assembly (Table 1). The orthogroup associated with the serine racemase (*SRR*) gene, which is involved in neurogenesis (Roychaudhuri et al. 2023), appeared to have undergone significant expansion in the *Sorex* shrews (Table 4). Other significantly expanded gene families in all Soricidae were associated with nervous system development genes and cellular response to stress and starvation, olfactory receptors, and the immune system (Fig. 2e). The gene families that underwent significant contractions shared between species were related to olfactory receptors and blood group system pathways (Fig. 2e).

### Episodic Diversifying Selection in Shrews

**Fig. 3.**
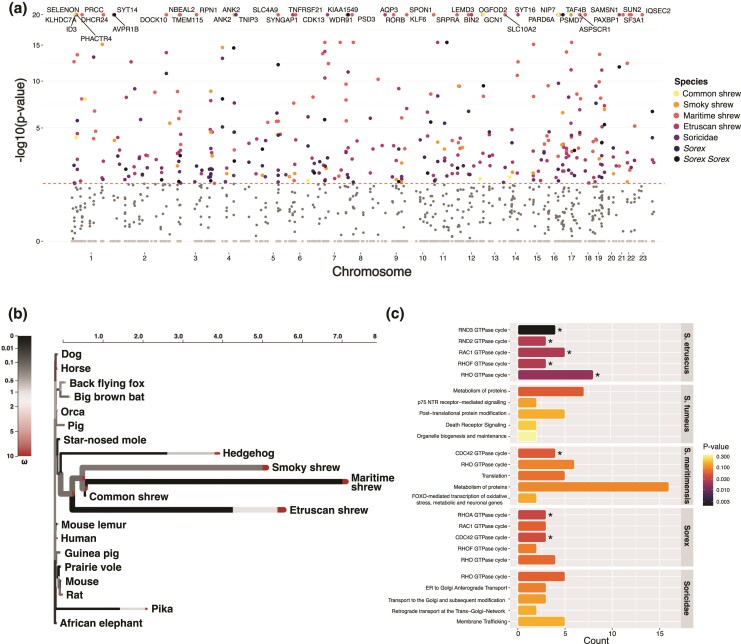
aBSREL (dN/dS) analysis results. (a) Manhattan plot of Bonferroni-Holm corrected *P*-values used as evidence for selection for all 533 single-copy genes from the foreground run mapped to the human (hg38) genome. Dark grey points represent genes with non-significant *P*-values for shrew nodes and species. Light grey points represent genes from all other mammal nodes and species. The red dotted line represents the 0.05 significance threshold. Top genes for shrew nodes and species are indicated by the respective name. (b) HyPhy Vision plot for the different omega (ω) rate distributions in each branch for the ANK2 gene. The color of the segments indicates the rate (ω), while the length of each segment represents the proportion of sites exhibiting that specific ω rate. Thicker branches highlight those identified as having undergone diversifying positive selection (corrected *P*-value < 0.05). (c) Top Reactome pathways for the positively selected genes from the dN/dS analysis in shrew nodes and species. Asterisk represents significant pathways (*P*-value < 0.05).

We calculated the ratio of non-synonymous to synonymous substitutions (dN/dS) using aBSREL to identify positive selection. Out of the 533 single-copy genes tested, nine were detected as being under positive selection in the common shrew, 97 in the maritime shrew, 31 in the smoky shrew, and 116 in the Etruscan shrew when the Soricidae common ancestor and all descendent branches were selected as the foreground branches (Fig. 3a, Table 3). A total of 62 genes were detected as being under positive selection in the Soricidae node, and 47 were detected as being selected for in the *Sorex* node (Fig. 3a, Table 3). Ninety-four percent of these genes had trees supporting shrews as a monphyletic group. In all shrew nodes and species, except for the common shrew, the ankyrin 2 (*ANK2*) gene, involved in making proteins found in the brain and heart, was under positive selection (Fig. 3b, Table 4). Other genes under selection included anoctamin 10 (*ANO10*), myosin IXB (*MYO9B*), and reticulon 4 receptor (*RTN4R*) which are all associated with the nervous system (Table 4). Top enriched pathways for positively selected genes were related to protein metabolism and various GTPases cycles (Fig. 3c).

## Discussion

Over the course of mammalian evolution, species have adapted to diverse ecological niches, evolving an array of specialized traits and behaviors. Shrews, in particular, have evolved unique phenotypes and abilities tailored to their various environments, including venomous saliva (Kita et al. 2004), echolocation (Tomasi 1979; Forsman and Malmquist 1988; Chai et al. 2020), hunting underwater (Mendes-Soares and Rychlik 2009), and reversible body size changes (Lázaro and Dechmann 2021). Soricidae are also known to have one of the shortest lifespans and fastest mass-specific metabolic rate of all mammals (Ochocińska and Taylor 2005). Here, we assembled two de novo shrew genomes and conducted comparative genomic analyses between four shrews and 16 other mammal species to test our prediction that shared genomic variants among shrew species are likely associated with unique phenotypes and abilities specific to shrews. We identified ARs, gene family expansion and contractions, as well as genes undergoing positive selection in Soricidae.

### Genome Evolution Underling Phenotypic Changes in Shrews

Shrews have poor vision which is mostly used to detect light intensity; in contrast, their olfactory, tactile, and acoustic senses are well developed (Churchfield 1990). Certain Nearctic shrew species, and the common shrew, have even developed echolocating abilities to navigate their surroundings (Gould 1969; Forsman and Malmquist 1988; Churchfield 1990). We found shared ARs in the coding region of the *CDH23* gene and 3′UTR region of the *OTOF* gene in the common and maritime shrews (Table 4). Both genes are involved in the auditory system and more specifically echolocation in mammals (Shen et al. 2012). Chai et al. (2020) found evidence of convergent evolution between the common shrew and other echolocating mammals for both these genes. The common shrew is the only species including in our analyses confirmed to echolocate, but our findings indicate the potential for such ability in the maritime shrew, as it is the most closely related species and shares ARs with the common shrew for the *CDH23* and *OTOF* genes. Echolocation is likely more widespread among shrews (i.e. *Blarina*) (Gleason et al. 2023), including other *Sorex* (Buchler 1976), thus detection in the maritime shrew is not unexpected and this might be a *Sorex*-wide trait.

Survival in cold climates involves species adopting strategies such as migration, hibernation, or entering a state of torpor. Shrews do not hibernate nor migrate long distances (Churchfield 1990). Their high metabolic rate prevents them from building up sufficient fat storage (Churchfield 1990); therefore, to survive through the winter, some shrew species have evolved a way to lower their energetical demand by undergoing reversible seasonal changes in body size and mass, known as Dehnel's phenomenon (Lázaro and Dechmann 2021). During the winter, brain mass can shrink around 20% in the common shrew and regrow up to 17% for the summer (Lázaro et al. 2018a). This has also been shown in the Etruscan shrew (Ray et al. 2020), and other *Sorex* shrews (e.g. *Sorex minutus*) (Bartkowska et al. 2008; Lázaro and Dechmann 2021). It remains uncertain if the maritime shrew and smoky shrew exhibit Dehnel's phenomenon, however both species belong to the *Sorex* genus and inhabit Northern climates, akin to other shrew species known to undergo these size changes.

We observed hundreds of ARs in shrews associated with nervous system development and axon guidance pathways (Fig. 1b), including specific genes such as *TUBB3*, *GAP43*, and *FGFR1* (Table 4). In all *Sorex* species, ARs were found in the 5′UTR regions of the *TUBB3* and *GAP43* genes. *TUBB3* plays a role in the regeneration of axons (Latremoliere et al. 2018), while *GAP43* is involved in neurite outgrowth and synaptic plasticity in the hippocampus (Aigner et al. 1995; Lee et al. 2023), a region of the brain known to shrink during the winter and regrow in the spring in the common shrew (Lázaro et al. 2018b). Furthermore, ARs were identified in the coding region of the *FGFR1* gene, which is also involved in hippocampal growth (Ohkubo et al. 2004). These findings were further supported by the gene family expansions. Gene families associated with the *SRR*, *PURA*, and *UGT8* genes, which are all involved with the brain and the nervous system (Table 4), had undergone significant expansion in all *Sorex* species. And in all Soricidae species, significantly expanded gene families were associated with SLITs and ROBOs pathways (Fig. 2e) which are involved in neocortical development, a region responsible for sight and hearing (Gonda et al. 2020). The pathway analysis further revealed that positively selected genes in shrew were related to Rho GTPase pathways (Fig. 3c). Rho GTPases, such as RhoA, Cdc42, and Rac1, are involved in neuronal development, neurodegeneration, and synaptic plasticity (Stankiewicz and Linseman 2014; Zhang et al. 2021). Finding such genes and pathways associated with the brain and nervous system consistently in all analyses and shared between all species, we hypothesize, is indicative of the genetic mechanisms involved in seasonal brain size plasticity.

Shrews might also undergo a metabolic shift from lipid to glucose as a fuel source to survive the winter. Work by Thomas et al. (2023) found evidence that lipid metabolite concentration decreased throughout the winter in the common shrew, possibly promoting carbohydrate metabolism during the harsh winter months. Thomas et al. (2023) found differentially expressed genes and pathways associated with insulin and cholesterol between shrew brain and liver samples across seasons. When consumed, carbohydrates breakdown into glucose, and insulin then regulates if it is used as a source of energy or stored (Dube et al. 2013). We identified ARs and positively selected genes in shrews associated with Rho GTPase pathways (supplementary fig. S2, Supplementary Material online, Fig. 3). Rho GTPase are also involved in metabolic homeostasis more specifically glucose metabolism (Møller et al. 2019). Furthermore, an AR was identified in the 3′UTR region of the *ADIPOR1* gene in *Sorex* (Table 4). *ADIPOR1* plays a crucial role in the regulation of glucose and lipid metabolism (Yamauchi et al. 2007). All shrew species shared an AR in the *GAPDH* coding region, which is involved in glycolysis, the process that breaks down glucose into energy (Table 4). Furthermore, gene families that underwent significant expansions were associated with cellular response to stress and starvation (Fig. 2e). These findings highlight the relationship between genomic variants unique to shrews and their distinctively fast metabolism and wintering strategy and offers candidate genes for further hypothesis testing (Table 4).

### Addressing Bias From Assembly and Annotation Differences

The divergent assembly strategies and genome quality statistics (Table 1) presented us with a natural study to compare the impact of reference genome that warrants comment. Assembly inconsistencies between genomes can lead to overestimation of genomic differences between taxa when conducting comparative genomic analyses (Denton et al. 2014). Our shrew assemblies consisted of three highly contiguous genomes (Etruscan, smoky, and common shrew), and one fragmented genome (maritime shrew). In the AR analysis, the maritime shrew and Etruscan shrew assemblies revealed a high number of ARs; however, we found no clear pattern between number of ARs and genome assembly quality or contiguity (supplementary fig. S3, Supplementary Material online). For good measure, we repeated the analysis to identify ARs in the African elephant (*Loxodonta africana*) and big brown bat (*Eptesicus fuscus*) and compared these values to the study done by Ferris et al. (2018). Our results were consistent with their work, with a significant lower number of ARs in the African elephant (*n* = 1,904) compared to the bat (*n* = 20,939; supplementary table S1, Supplementary Material online). Thus, the AR analysis appears to not be impacted by highly fragmented assemblies like the maritime shrew, likely due to the bioinformatic approach of mapping short fragments.

The maritime shrew had fewer genes assigned to orthogroups and more species-specific orthogroups compared to all other shrews. Here, this is likely due to the maritime shrew's lower genome contiguity and not having a standardized RefSeq annotation like all other species. Depending on the sequencing and assembly method, de novo genome assemblies can be fragmented resulting in inaccurate structure and number of predicted genes (Denton et al. 2014). However, despite detecting considerably more orthogroups in the maritime shrew, the number of duplicated regions and genes under positive selection did not deviate significantly from the Etruscan shrew (Table 3), suggesting that none of our metrics were drastically impacted by fragmentation. Still, to avoid biases due to assembly and annotation differences between species, we focused on overall trends found in Soricidae and *Sorex* instead of species-specific trends. This minimizes overestimation of ARs, gene duplication event, or gene family expansion/contraction and positively selected genes identified in shrews. Overall, our study found shrew-specific genomic variants in genes associated with the nervous, auditory, metabolic, and olfactory systems, most of which can be linked to unique phenotypes or traits in shrews.

## Materials and Methods

### Sampling and Sequencing

A single smoky shrew (*S. fumeus*) was live captured from Peterborough, Ontario (Animal Care Certificate no. 26234). Heart, liver, brain, and tail tissue were collected immediately after euthanasia and frozen. DNA was extracted from the heart and liver using the *MagAttract HMW DNA Kit* from QIAGEN. Pooled DNA extracts were sent to The Centre for Applied Genomics (TCAG) in Toronto, Ontario, Canada, to sequence five HiFi cells on the PacBio Sequel II instrument. Tissue samples were sent to Phase Genomics (Seattle, Washington) to generate a Hi-C library using the Phase Genomics Proximo Animal kit version 4.0.

Genomic sequencing of the maritime shrew was undertaken as part of the CanSeq150 program (https://www.cgen.ca/canseq150). A single maritime shrew (*S. maritimensis*) was live captured and euthanized following the guidelines of the Canadian Council on Animal Care—see Dawe et al. (2009). Liver tissue was collected immediately and frozen. Genomic DNA was extracted from frozen tissues using a standard proteinase K-phenol-chloroform protocol (Sambrook et al. 1989). DNA and tissues were stored at −80°, and DNA extracts from this maritime shrew were sent to TCAG for sequencing. Short-read (SR) libraries were generated using PCR-free preparation and sequenced on two lanes on the Illumina HiSeqX instrument and with 150 bp paired-end reads. Linked reads (LR) were prepared using the 10× genome library after selecting fragments > 15 kb using BluePippin. The LR were sequenced on one Illumina HiSeq X Lane with 150 bp paired-end reads.

### Genome Assemblies and Annotation

We assembled the smoky shrew reference genome following the updated Vertebrate Genome Pipeline (Rhie et al. 2021). The HiFi bam files were filtered using bamtools version 2.5.1 (Barnett et al. 2011) (rq ≥ 0.99) and converted to fastq files using samtools version 1.12 (Danecek et al. 2021) via the *fastq* flag. We trimmed residual HiFi adaptor sequences using cutadapt version 3.4 (Martin 2011). The primary assembly was constructed using hifiasm version 0.16.1-r375 (Cheng et al. 2021, 2022) allowing for the integration of the Hi-C reads. We used the default level of purge duplication of three for non-trio assembly. We ran the hifiasm python module on 32 threads with 228 GB of RAM. We used Merqury version 1.3 (Rhie et al. 2020) with meryl from Canu version 2.2 (Koren et al. 2017) to assess genome assembly quality via k-mer copy number analysis.

For the maritime shrew genome, we used BBMap version 35.8 (Bushnell 2022) to remove adapters and trim low-quality data from the fastq files. Kmergenie version 1.7048 (Chikhi and Medvedev 2014) was used to find the k-mer size for downstream analysis. The genome was assembled using a tiered approach: (i) using only the SR data with w2rap-contigger with -K set to 144 (Clavijo 2021) and (ii) with only the 10X LR data using Supernova version 2.1.1 (Weisenfeld et al. 2018) using the --maxreads filter set to “all”. The pseudohap2 style was selected for the assembly output. Following these assemblies, both SR and LR assembly versions were merged via quickmerge version 0.3 (Chakraborty et al. 2016) with the LR as the backbone and the default parameters. Scaff10x version 5.0 (Ning et al. n.d) was used to further polish the maritime shrew genome.

Nuclear genome completeness was assessed for both assemblies using Benchmarking universal single-copy orthologues (BUSCO) version 3.0.2 (Simão et al. 2015) by comparing the genomes to highly conserved genes in mammals. The smoky shrew mitochondrial genome was assembled using the PacBio subreads and the mitochondrial genome of a previous smoky shrew assembly (NCBI accession: GCA_026122425.1) (Cossette et al. 2023) as a backbone with MitoHifi version 3.2 (Uliano-Silva et al. 2023). The mitochondrial genome for the maritime shrew was assembled using MitoZ (Meng et al. 2019) with the function “mitoz all” on the fastq reads and parameters --genetic_code 2 --clade Chordata --kmers_megahit 59 63 79 99 119 141 --requiring_taxa Chordata and was manually curated.

The smoky shrew genome annotation was generated by NCBI using their eukaryotic genome annotation pipeline that integrated RNAseq data (SRA RNA-Seq accession: SRX20204431, SRX20204430) from Cossette et al. (2023). We generated the maritime shrew annotation in-house via GenSAS version 6.0 (Humann et al. 2019) as it was too fragmented for the NCBI pipeline. Repeat regions were identified using RepeatModeler version 2.0.1 (Smit and Hubley 2023) and RepeatMasker version 4.1.1 Smit and Hubley (2023). Here, we used the smoky shrew liver and heart RNA reads from Cossette et al. (2023), as we did not have RNA data for the maritime shrew, and mapped them to the maritime shrew genome using HISAT2 version 2.2.1 (Kim et al. 2019). The resulting BAM files were used by Augustus version S3.4.0 (Stanke et al. 2006) for gene prediction. The NCBI refseq vertebrate-mammalian protein database available on GenSAS was aligned to the genome using DIAMOND version 2.0.11 (Buchfink et al. 2015). Augustus and DIAMOND were also run using the common shrew, mSorAra2.pri (GenBank accession: GCF_027595985.1), protein fasta file. EVidenceModeler version 1.1.1 (Haas et al. 2008) was used to generate a consensus gene set using the Augustus (1× weight) and DIAMOND (5× weight) outputs. Gene function was assigned to our gene consensus model with DIAMOND and InterProScan version 5.53-87.0 (Jones et al. 2014)

### Detection of ARs in Shrew Genomes

We generated a multiple alignment file (MAF) consisting of 20 mammalian genome assemblies to identify ARs in the four shrew species. We downloaded the pairwise syntenic net alignment files for the 16 mammals, including the common shrew, against the human (hg38) genome (supplementary table S2, Supplementary Material online) from the UCSC database (Kent et al. 2002). Using LASTZ version 1.04.03 (Harris 2007), we generated pairwise alignments against the hg38 genome for the smoky, maritime, and Etruscan shrew genomes. The LASTZ run parameters were set to K = 3,000, L = 3,000, Y = 9,400, E = 30, H = 2,000, and O = 400 based on UCSC's hg38 100-way conservation parameters for their common shrew alignment. The alignments for each species were chained with the chainMinScore = 3000 and linearGap = medium options and subsequently netted using kentutils tools version 401 (Kent 2020). Our resulting pairwise alignments for each shrew species were then aligned to the other mammal pairwise alignments to create one MAF using the roast function from MULTIZ version 11.2 (Blanchette et al. 2004) and the tree topology in supplementary fig. S1b, Supplementary Material online, supported by Murphy et al. (2001) and Meredith et al. (2011).

The MAF was filtered to only keep alignments to the 22 main autosomal chromosomes of the hg38 genome. We used the msa_view function from the PHAST package version 1.5 (Hubisz et al. 2011) to extract 4-fold degenerate (4D) sites from the non-shrew species in our alignments and based on the human annotations (NCBI accession: GCA_000001405.15) from which we only kept coding sequences. The output was used to estimate a non-conserved phylogenetic model using phyloFit with the substitution model REV and the EM algorithm option. The PhastCons function from RPHAST version 1.6.11 (Hubisz et al. 2011) was used to identify conserved regions in the non-shrew species with the parameters set to expected.length = 45, target.coverage = 0.3, rho = 0.31, and viterbi = TRUE. We filtered the predicted conserved regions to sites that aligned to all the shrew species and at least 18 species in total. We regularized the length of the conserved regions to 50 bp regions to simplify the likelihood ratio tests. PhyloP was run using the acceleration (ACC) mode with the --features option to identify ARs within the predicted conserved regions for each of our shrew species. This was done by running phyloP sequentially on each shrew terminal branch using the full species alignment. Nonparametric simulations were run to calculate empirical *P*-values by generating 100,000 synthetic alignments and running phyloP on these alignments to obtain a null distribution of log likelihood ratios. Statistically significant ARs were defined with a false discovery rate threshold of 5%. Genes overlapping these ARs were identified using bedtools version 2.30.0 (Quinlan and Hall 2010) and the hg38 genome annotations.

### Gene Family Size Evolution

We identified orthogroups among proteins sequences from all 20 mammal genomes (supplementary fig. S1b, Supplementary Material online, supplementary table S2, Supplementary Material online) to identify gene duplication events. Here, an orthogroup is a set of genes that have descended from a single ancestral gene in the last common ancestor to our species of interest and all other mammalian genomes in the set (Emms and Kelly 2019). We downloaded the protein files from NCBI RefSeq for each species (supplementary table S2, Supplementary Material online), except the maritime shrew for which we used our GenSAS annotation. We further filtered the data to keep the longest transcript variant per gene in each species’ file to minimize run time and increase accuracy. OrthoFinder version 2.5.4 (Emms and Kelly 2019) with the parameters -M msa, -S diamond, -A mafft, -z, -T fasttree was used to obtain 20,489 orthogroups and identify duplicated genes. We then used CAFE version 4.2 (De Bie et al. 2006) with a multi-lambda model to analyze the evolution of orthogroup sizes and used an error estimation model to account for genome assembly errors. A Monte Carlo resampling procedure was applied to each branch and node to compute family-wide *P*-values. For *P* ≤ 0.01, the Viterbi method was used to calculate exact *P*-values and identify gene families that have experienced significant expansion (*P* ≤ 0.01) (De Bie et al. 2006).

### Non-synonymous to Synonymous Rate Ratio

We used an adaptive branch site random effects likelihood (aBSREL) model (Smith et al. 2015) with the HyPhy package version 2.5.49 (Pond et al. 2005) to identify possible episodic diversifying selection in shrews by calculating the ratio of non-synonymous to synonymous substitutions (dN/dS). The analysis was performed on the 533 single-copy genes identified by OrthoFinder. Multiple sequence alignment for each protein was converted to sequence codon alignments using PAL2NAL version 14.1 (Suyama et al. 2006) to input in aBSREL using the coding sequence available on RefSeq (supplementary table S2, Supplementary Material online). We examined the individual gene trees for each single-copy gene alignment and found that 80% of these trees supported shrews as a monophyletic group. We used the consensus species tree output from the OrthoFinder analysis, which is based on approximately 20,000 orthogroups and also includes shrews as a monophyletic group, as the input tree for all aBSREL runs. We ran the aBSREL analysis twice to estimate dN/dS, first (i) with the branch leading to the common ancestor of the shrews and all its descendent branches selected as the foreground; and (ii) with no foreground branches specification and all branches tested for positive selection. An R script (Jarva 2023) was used to parse and extract data from the nested json file outputs.

### Gene Pathway Analysis

Gene and protein summaries were manually retrieved from UniProt (UniProt Consortium 2023) and NCBI (National Center for Biotechnology Information (NCBI), 1988). Genes that were associated with ARs, duplications events, statistically significant family size changes, and positive selection in shrews were linked to the corresponding human ortholog gene ID when possible, to input in DAVID, the Database for Annotation, Visualization and Integrated Discovery (Huang et al. 2009; Sherman et al. 2022), to identify gene pathways for each analysis.

## Supplementary Material

evae225_Supplementary_Data

## Data Availability

Smoky shrew genome and raw sequence reads deposited in GenBank/NCBI under project: PRJNA826195. Maritime shrew genome and raw sequence reads deposited in GenBank/NCBI under project: PRJNA956518. Scripts are uploaded on GitLab: https://gitlab.com/WiDGeT_TrentU/graduate_theses.git.

## References

[evae225-B1] Aigner L, Arber S, Kapfhammer JP, Laux T, Schneider C, Botteri F, Brenner H-R, Caroni P. Overexpression of the neural growth-associated protein GAP-43 induces nerve sprouting in the adult nervous system of transgenic mice. Cell. 1995:83(2):269–278. 10.1016/0092-8674(95)90168-X. 7585944

[evae225-B2] Barnett DW, Garrison EK, Quinlan AR, Strömberg MP, Marth GT. BamTools: a C++ API and toolkit for analyzing and managing BAM files. Bioinformatics. 2011:27(12):1691–1692. 10.1093/bioinformatics/btr174. 21493652 PMC3106182

[evae225-B3] Bartkowska K, Djavadian RL, Taylor JRE, Turlejski K. Generation recruitment and death of brain cells throughout the life cycle of Sorex shrews (Lipotyphla). European Journal of Neuroscience. 2008:27(7):1710–1721. 10.1111/j.1460-9568.2008.06133.x. 18380668

[evae225-B4] Bi X, Zhou L, Zhang J-J, Feng S, Hu M, Cooper DN, Lin J, Li J, Wu D-D, Zhang G. Lineage-specific accelerated sequences underlying primate evolution. Sci Adv. 2023:9(22):eadc9507. 10.1126/sciadv.adc9507. 37262186 PMC10413682

[evae225-B5] Blanchette M, Kent WJ, Riemer C, Elnitski L, Smit AFA, Roskin KM, Baertsch R, Rosenbloom K, Clawson H, Green ED, et al Aligning multiple genomic sequences with the threaded blockset aligner. Genome Res. 2004:14(4):708–715. 10.1101/gr.1933104. 15060014 PMC383317

[evae225-B6] Buchfink B, Xie C, Huson DH. Fast and sensitive protein alignment using DIAMOND. Nat Methods. 2015:12(1):59–60. 10.1038/nmeth.3176. 25402007

[evae225-B7] Buchler ER . The use of echolocation by the wandering shrew (*Sorex vagrans*). Anim Behav. 1976:24(4):858–873. 10.1016/S0003-3472(76)80016-4.

[evae225-B8] Burgin CJ, Colella JP, Kahn PL, Upham NS. How many species of mammals are there? J Mammal. 2018:99(1):1–14. 10.1093/jmammal/gyx147.

[evae225-B9] Bushnell B . BBTools. 2022. https://sourceforge.net/projects/bbmap/

[evae225-B10] Chai S, Tian R, Rong X, Li G, Chen B, Ren W, Xu S, Yang G. Evidence of echolocation in the common shrew from molecular convergence with other echolocating mammals. Zool Stud. 2020:59:e4. 10.6620/ZS.2020.59-4. 32494297 PMC7262541

[evae225-B11] Chakraborty M, Baldwin-Brown JG, Long AD, Emerson JJ. Contiguous and accurate de novo assembly of metazoan genomes with modest long read coverage. Nucleic Acids Res. 2016:44(1):e147. 10.1093/nar/gkw654. 27458204 PMC5100563

[evae225-B12] Cheng H, Concepcion GT, Feng X, Zhang H, Li H. Haplotype-resolved de novo assembly using phased assembly graphs with hifiasm. Nat Methods. 2021:18(2):170–175. 10.1038/s41592-020-01056-5. 33526886 PMC7961889

[evae225-B13] Cheng H, Jarvis ED, Fedrigo O, Koepfli K-P, Urban L, Gemmell NJ, Li H. Haplotype-resolved assembly of diploid genomes without parental data. Nat Biotechnol. 2022:40(9):1332–1335. 10.1038/s41587-022-01261-x. 35332338 PMC9464699

[evae225-B14] Chikhi R, Medvedev P. Informed and automated k-mer size selection for genome assembly. Bioinformatics. 2014:30(1):31–37. 10.1093/bioinformatics/btt310. 23732276

[evae225-B15] Christmas MJ, Kaplow IM, Genereux DP, Dong MX, Hughes GM, Li X, Sullivan PF, Hindle AG, Andrews G, Armstrong JC, et al Evolutionary constraint and innovation across hundreds of placental mammals. Science. 2023:380(6643):eabn3943. 10.1126/science.abn3943. 37104599 PMC10250106

[evae225-B16] Chrysanthou A, Ververis A, Christodoulou K. ANO10 function in health and disease. The Cerebellum. 2023:22(3):447–467. 10.1007/s12311-022-01395-3. 35648332 PMC10126014

[evae225-B17] Churchfield S . The natural history of shrews. Ithaca (NY): Cornell University Press; 1990.

[evae225-B18] Clavijo BJ . w2rap-contigger. 2021. https://github.com/bioinfologics/w2rap-contigger

[evae225-B19] Cossette M, Stewart DT, Haghani A, Zoller JA, Shafer ABA, Horvath S. Epigenetics and island-mainland divergence in an insectivorous small mammal. Mol Ecol. 2023:32(1):152–166. 10.1111/mec.16735. 36226847

[evae225-B20] Damas J, Corbo M, Kim J, Turner-Maier J, Farré M, Larkin DM, Ryder OA, Steiner C, Houck ML, Hall S, et al Evolution of the ancestral mammalian karyotype and syntenic regions. Proc Natl Acad Sci U S A. 2022:119(40):e2209139119. 10.1073/pnas.2209139119. 36161960 PMC9550189

[evae225-B21] Damas J, Corbo M, Lewin HA. Vertebrate chromosome evolution. Annu Rev Anim Biosci. 2021:9(1):1–27. 10.1146/annurev-animal-020518-114924. 33186504

[evae225-B22] Danecek P, Bonfield JK, Liddle J, Marshall J, Ohan V, Pollard MO, Whitwham A, Keane T, McCarthy SA, Davies RM, et al Twelve years of SAMtools and BCFtools. GigaScience. 2021:10(2):giab008. 10.1093/gigascience/giab008. 33590861 PMC7931819

[evae225-B23] Dawe KL, Shafer ABA, Herman TB, Stewart DT. Diffusion of nuclear and mitochondrial genes across a zone of secondary contact in the maritime shrew, *Sorex maritimensis*: implications for the conservation of a Canadian endemic mammal. Conserv Genet. 2009:10(4):851–857. 10.1007/s10592-008-9645-7.

[evae225-B24] De Bie T, Cristianini N, Demuth JP, Hahn MW. CAFE: a computational tool for the study of gene family evolution. Bioinformatics. 2006:22(10):1269–1271. 10.1093/bioinformatics/btl097. 16543274

[evae225-B25] Denton JF, Lugo-Martinez J, Tucker AE, Schrider DR, Warren WC, Hahn MW. Extensive error in the number of genes inferred from draft genome assemblies. PLoS Comput Biol. 2014:10(12):e1003998. 10.1371/journal.pcbi.1003998. 25474019 PMC4256071

[evae225-B26] Dube S, Errazuriz I, Cobelli C, Basu R, Basu A. Assessment of insulin action on carbohydrate metabolism: physiological and non-physiological methods. Diabet Med. 2013:30(6):664–670. 10.1111/dme.12189. 23683103 PMC3662485

[evae225-B27] Dziȩgiel P, Owczarek T, Plaz̀uk E, Gomułkiewicz A, Majchrzak M, Podhorska-Okołów M, Driouch K, Lidereau R, Ugorski M. Ceramide galactosyltransferase (UGT8) is a molecular marker of breast cancer malignancy and lung metastases. Br J Cancer. 2010:103(4):524–531. 10.1038/sj.bjc.6605750. 20648017 PMC2939773

[evae225-B28] Emms DM, Kelly S. OrthoFinder: phylogenetic orthology inference for comparative genomics. Genome Biol. 2019:20(1):238. 10.1186/s13059-019-1832-y. 31727128 PMC6857279

[evae225-B29] Ferris E, Abegglen LM, Schiffman JD, Gregg C. Accelerated evolution in distinctive species reveals candidate elements for clinically relevant traits, including mutation and cancer resistance. Cell Rep. 2018:22(10):2742–2755. 10.1016/j.celrep.2018.02.008. 29514101 PMC6294302

[evae225-B30] Forsman KA, Malmquist MG. Evidence for echolocation in the common shrew, *Sorex araneus*. J Zool. 1988:216(4):655–662. 10.1111/j.1469-7998.1988.tb02463.x.

[evae225-B31] Fumagalli L, Taberlet P, Stewart DT, Gielly L, Hausser J, Vogel P. Molecular phylogeny and evolution of Sorex shrews (Soricidae: Insectivora) inferred from mitochondrial DNA sequence data. Mol Phylogenet Evol. 1999:11(2):222–235. 10.1006/mpev.1998.0568. 10191067

[evae225-B32] George SB . Evolution and historical biogeography of soricine shrews. Syst Zool. 1986:35(2):153–162. 10.2307/2413427.

[evae225-B33] George SB . Systematics, historical biogeography, and evolution of the genus *Sorex*. J Mammal. 1988:69(3):443–461. 10.2307/1381337.

[evae225-B34] Gleason ME, Eddington VM, Kloepper LN. Acoustic behavior in the northern short-tailed shrew (*Blarina brevicauda*): ultrasonic click production in a novel environment. J Acoust Soc Am. 2023:154(1):411–417. 10.1121/10.0020071. 37477634

[evae225-B35] Gonda Y, Namba T, Hanashima C. Beyond axon guidance: roles of slit-robo signaling in neocortical formation. Front Cell Dev Biol. 2020:8:607415. 10.3389/fcell.2020.607415. 33425915 PMC7785817

[evae225-B36] Gould E . Communication in three genera of shrews (Soricidae): *Suncus*, *Blarina*, and *Cryptotis*. Commun Behav Biol. 1969:3:11–31.

[evae225-B37] Haas BJ, Salzberg SL, Zhu W, Pertea M, Allen JE, Orvis J, White O, Buell CR, Wortman JR. Automated eukaryotic gene structure annotation using EVidenceModeler and the program to assemble spliced alignments. Genome Biol. 2008:9(1):R7. 10.1186/gb-2008-9-1-r7. 18190707 PMC2395244

[evae225-B38] Harris RS . Improved pairwise alignment of genomic DNA. University Park (PA): The Pennsylvania State University; 2007.

[evae225-B39] Helsen J, Voordeckers K, Vanderwaeren L, Santermans T, Tsontaki M, Verstrepen KJ, Jelier R. Gene loss predictably drives evolutionary adaptation. Mol Biol Evol. 2020:37(10):2989–3002. 10.1093/molbev/msaa172. 32658971 PMC7530610

[evae225-B40] Huang DW, Sherman BT, Lempicki RA. Systematic and integrative analysis of large gene lists using DAVID bioinformatics resources. Nat Protoc. 2009:4(1):44–57. 10.1038/nprot.2008.211. 19131956

[evae225-B41] Hubisz MJ, Pollard KS. Exploring the genesis and functions of human accelerated regions sheds light on their role in human evolution. Curr Opin Genet Dev. 2014:29:15–21. 10.1016/j.gde.2014.07.005. 25156517

[evae225-B42] Hubisz MJ, Pollard KS, Siepel A. PHAST and RPHAST: phylogenetic analysis with space/time models. Brief Bioinform. 2011:12(1):41–51. 10.1093/bib/bbq072. 21278375 PMC3030812

[evae225-B43] Humann JL, Lee T, Ficklin S, Main D. Structural and functional annotation of eukaryotic genomes with GenSAS. Methods Mol Biol. 2019:1962:29–51. 10.1007/978-1-4939-9173-0_3. 31020553

[evae225-B44] Hutterer R . Order soricomorpha. In: Wilson DE, Reeder DM, editors. Mammal species of the world: a taxonomic and geographic reference. 3rd ed. Baltimore, Maryland: The Johns Hopkins University Press; 2005. p. 220–311.

[evae225-B45] Jacquet S, Culbertson M, Zhang C, El Filali A, De La Myre Mory C, Pons J-B, Filippi-Codaccioni O, Lauterbur ME, Ngoubangoye B, Duhayer J, et al Adaptive duplication and genetic diversification of protein kinase R contribute to the specificity of bat–virus interactions. Sci Adv. 2022:8(47):eadd7540. 10.1126/sciadv.add7540. 36417524 PMC9683710

[evae225-B46] Jarva T. 2023. parse_all_absREL_json. [accessed 2023 October 18]. https://github.com/stupornova33/parse_absREL_json/blob/main/parse_all_absREL_json.

[evae225-B47] Jones P, Binns D, Chang HY, Fraser M, Li W, McAnulla C, McWilliam H, Maslen J, Mitchell A, Nuka G, et al InterProScan 5: genome-scale protein function classification. Bioinformatics. 2014:30(9):1236–1240. 10.1093/bioinformatics/btu031. 24451626 PMC3998142

[evae225-B48] Kent WJ, Sugnet CW, Furey TS, Roskin KM, Pringle TH, Zahler AM, Haussler D. The human genome browser at UCSC. Genome Res. 2002:12(6):996–1006. 10.1101/gr.229102. 12045153 PMC186604

[evae225-B49] Kent WJ. 2020. UCSC Genome Browser. [accessed 2023 January 19]. https://github.com/ucscGenomeBrowser/kent.

[evae225-B50] Khalili K, Del Valle L, Muralidharan V, Gault WJ, Darbinian N, Otte J, Meier E, Johnson EM, Daniel DC, Kinoshita Y, et al Purα is essential for postnatal brain development and developmentally coupled cellular proliferation as revealed by genetic inactivation in the mouse. Mol Cell Biol. 2003:23(19):6857–6875. 10.1128/MCB.23.19.6857-6875.2003. 12972605 PMC193944

[evae225-B51] Kim D, Paggi JM, Park C, Bennett C, Salzberg SL. Graph-based genome alignment and genotyping with HISAT2 and HISAT-genotype. Nat Biotechnol. 2019:37(8):907–915. 10.1038/s41587-019-0201-4. 31375807 PMC7605509

[evae225-B52] Kimura H, Fujita Y, Kawabata T, Ishizuka K, Wang C, Iwayama Y, Okahisa Y, Kushima I, Morikawa M, Uno Y, et al A novel rare variant R292H in RTN4R affects growth cone formation and possibly contributes to schizophrenia susceptibility. Transl Psychiatry. 2017:7(8):e1214. 10.1038/tp.2017.170. 28892071 PMC5611737

[evae225-B53] Kita M, Nakamura Y, Okumura Y, Ohdachi SD, Oba Y, Yoshikuni M, Kido H, Uemura D. Blarina toxin, a mammalian lethal venom from the short-tailed shrew *Blarina brevicauda*: isolation and characterization. Proc Natl Acad Sci U S A. 2004:101(20):7542–7547. 10.1073/pnas.0402517101. 15136743 PMC419642

[evae225-B54] Koren S, Walenz BP, Berlin K, Miller JR, Bergman NH, Phillippy AM. Canu: scalable and accurate long-read assembly via adaptive k-mer weighting and repeat separation. Genome Res. 2017:27(5):722–736. 10.1101/gr.215087.116. 28298431 PMC5411767

[evae225-B55] Latremoliere A, Cheng L, DeLisle M, Wu C, Chew S, Hutchinson EB, Sheridan A, Alexandre C, Latremoliere F, Sheu S-H, et al Neuronal-specific TUBB3 is not required for normal neuronal function but is essential for timely axon regeneration. Cell Rep. 2018:24(7):1865–1879.e9. 10.1016/j.celrep.2018.07.029. 30110642 PMC6155462

[evae225-B56] Lázaro J, Dechmann DKN. Dehnel's phenomenon. Curr Biol. 2021:31(10):R463–R465. 10.1016/j.cub.2021.04.006. 34033763

[evae225-B57] Lázaro J, Hertel M, LaPoint S, Wikelski M, Stiehler M, Dechmann DKN. Cognitive skills of common shrews (*Sorex araneus*) vary with seasonal changes in skull size and brain mass. J Exp Biol. 2018a:221(Pt 2):jeb166595. 10.1242/jeb.166595. 29170257

[evae225-B58] Lázaro J, Hertel M, Sherwood CC, Muturi M, Dechmann DKN. Profound seasonal changes in brain size and architecture in the common shrew. Brain Struct Funct. 2018b:223(6):2823–2840. 10.1007/s00429-018-1666-5. 29663134 PMC5995987

[evae225-B59] Lee YJ, Jeong YJ, Kang EJ, Kang BS, Lee SH, Kim YJ, Kang SS, Suh SW, Ahn EH. GAP-43 closely interacts with BDNF in hippocampal neurons and is associated with Alzheimer's disease progression. Front Mol Neurosci. 2023:16:1150399 10.3389/fnmol.2023.1150399. 37143467 PMC10152972

[evae225-B60] Levchenko A, Kanapin A, Samsonova A, Gainetdinov RR. Human accelerated regions and other human-specific sequence variations in the context of evolution and their relevance for brain development. Genome Biol Evol. 2018:10(1):166–188. 10.1093/gbe/evx240. 29149249 PMC5767953

[evae225-B61] Li R, Meng M, Chen Y, Pan T, Li Y, Deng Y, Zhang R, Tian R, Xu W, Zheng X, et al ATP-citrate lyase controls endothelial gluco-lipogenic metabolism and vascular inflammation in sepsis-associated organ injury. Cell Death Dis. 2023:14(7):401. 10.1038/s41419-023-05932-8. 37414769 PMC10325983

[evae225-B62] Long H, Zhu X, Yang P, Gao Q, Chen Y, Ma L. Myo9b and RICS modulate dendritic morphology of cortical neurons. Cerebral Cortex. 2013:23(1):71–79. 10.1093/cercor/bhr378. 22250289

[evae225-B63] Magadum S, Banerjee U, Murugan P, Gangapur D, Ravikesavan R. Gene duplication as a major force in evolution. J Genet. 2013:92(1):155–161. 10.1007/s12041-013-0212-8. 23640422

[evae225-B64] Martin M . Cutadapt removes adapter sequences from high-throughput sequencing reads. EMBnet J. 2011:17(1):10. 10.14806/ej.17.1.200.

[evae225-B65] Mendes-Soares H, Rychlik L. Differences in swimming and diving abilities between two sympatric species of water shrews: *Neomys anomalus* and *Neomys fodiens* (Soricidae). J Ethol. 2009:27(3):317–325. 10.1007/s10164-008-0122-z.

[evae225-B66] Meng G, Li Y, Yang C, Liu S. MitoZ: a toolkit for animal mitochondrial genome assembly, annotation and visualization. Nucleic Acids Res. 2019:47(11):e63. 10.1093/nar/gkz173. 30864657 PMC6582343

[evae225-B67] Meredith RW, Janečka JE, Gatesy J, Ryder OA, Fisher CA, Teeling EC, Goodbla A, Eizirik E, Simão TLL, Stadler T, et al Impacts of the cretaceous terrestrial revolution and KPg extinction on mammal diversification. Science. 2011:334(6055):521–524. 10.1126/science.1211028. 21940861

[evae225-B68] Møller LLV, Klip A, Sylow L. Rho GTPases—emerging regulators of glucose homeostasis and metabolic health. Cells. 2019:8(5):434. 10.3390/cells8050434. 31075957 PMC6562660

[evae225-B69] Murphy WJ, Eizirik E, Johnson WE, Zhang YP, Ryder OA, O’Brien SJ. Molecular phylogenetics and the origins of placental mammals. Nature. 2001:409(6820):614–618. 10.1038/35054550. 11214319

[evae225-B70] National Center for Biotechnology Information (NCBI) . 1988. National Library of Medicine (US), National Center for Biotechnology Information. [accessed 2023 October 7]. https://www.ncbi.nlm.nih.gov/.

[evae225-B71] Ning Z, Giordano F, Harry E. n.d. Scaff10X. [accessed 2021 August 9]. https://github.com/wtsi-hpag/Scaff10X.

[evae225-B72] Ochocińska D, Taylor JRE. Living at the physiological limits: field and maximum metabolic rates of the common shrew (*Sorex araneus*). Physiol Biochem Zool. 2005:78(5):808–818. 10.1086/431190. 16096983

[evae225-B73] Ohkubo Y, Uchida AO, Shin D, Partanen J, Vaccarino FM. Fibroblast growth factor receptor 1 is required for the proliferation of hippocampal progenitor cells and for hippocampal growth in mouse. J Neurosci. 2004:24(27):6057–6069. 10.1523/JNEUROSCI.1140-04.2004. 15240797 PMC6729672

[evae225-B74] Okur V, Cho MT, Henderson L, Retterer K, Schneider M, Sattler S, Niyazov D, Azage M, Smith S, Picker J, et al De novo mutations in CSNK2A1 are associated with neurodevelopmental abnormalities and dysmorphic features. Hum Genet. 2016:135(7):699–705. 10.1007/s00439-016-1661-y. 27048600

[evae225-B75] Palma F, Di Holme RH, Bryda EC, Belyantseva IA, Pellegrino R, Kachar B, Steel KP, Noben-Trauth K. Mutations in Cdh23, encoding a new type of cadherin, cause stereocilia disorganization in waltzer, the mouse model for Usher syndrome type 1D. Nat Genet. 2001:27(1):103–107. 10.1038/83660. 11138008

[evae225-B76] Pollard KS, Salama SR, King B, Kern AD, Dreszer T, Katzman S, Siepel A, Pedersen JS, Bejerano G, Baertsch R, et al Forces shaping the fastest evolving regions in the human genome. PLoS Genet. 2006a:2(10):e168. 10.1371/journal.pgen.0020168. 17040131 PMC1599772

[evae225-B77] Pollard KS, Salama SR, Lambert N, Lambot M-A, Coppens S, Pedersen JS, Katzman S, King B, Onodera C, Siepel A, et al An RNA gene expressed during cortical development evolved rapidly in humans. Nature. 2006b:443(7108):167–172. 10.1038/nature05113. 16915236

[evae225-B78] Pond SLK, Frost SDW, Muse SV. HyPhy: hypothesis testing using phylogenies. Bioinformatics. 2005:21(5):676–679. 10.1093/bioinformatics/bti079. 15509596

[evae225-B79] Ponting CP . Biological function in the twilight zone of sequence conservation. BMC Biol. 2017:15(1):71. 10.1186/s12915-017-0411-5. 28814299 PMC5558704

[evae225-B80] Quinlan AR, Hall IM. BEDTools: a flexible suite of utilities for comparing genomic features. Bioinformatics. 2010:26(6):841–842. 10.1093/bioinformatics/btq033. 20110278 PMC2832824

[evae225-B81] Ray S, Li M, Koch SP, Mueller S, Boehm-Sturm P, Wang H, Brecht M, Naumann RK. Seasonal plasticity in the adult somatosensory cortex. Proc Natl Acad Sci U S A. 2020:117(50):32136–32144. 10.1073/pnas.1922888117. 33257560 PMC7749348

[evae225-B82] Reinhard JR, Kriz A, Galic M, Angliker N, Rajalu M, Vogt KE, Ruegg MA. The calcium sensor Copine-6 regulates spine structural plasticity and learning and memory. Nat Commun. 2016:7(1):11613. 10.1038/ncomms11613. 27194588 PMC4874034

[evae225-B83] Reumer JWF . Speciation and evolution in the Soricidae (Mammalia: Insectivora) in relation with the paleoclimate. Revue Suisse de Zoologie. 1989:96:81–90. 10.5962/bhl.part.117758.

[evae225-B84] Rhie A, McCarthy SA, Fedrigo O, Damas J, Formenti G, Koren S, Uliano-Silva M, Chow W, Fungtammasan A, Kim J, et al Towards complete and error-free genome assemblies of all vertebrate species. Nature. 2021:592(7856):737–746. 10.1038/s41586-021-03451-0. 33911273 PMC8081667

[evae225-B85] Rhie A, Walenz BP, Koren S, Phillippy AM. Merqury: reference-free quality, completeness, and phasing assessment for genome assemblies. Genome Biol. 2020:21(1):245. 10.1186/s13059-020-02134-9. 32928274 PMC7488777

[evae225-B86] Roux I, Safieddine S, Nouvian R, Grati M, Simmler M-C, Bahloul A, Perfettini I, Le Gall M, Rostaing P, Hamard G, et al Otoferlin, defective in a human deafness form, is essential for exocytosis at the auditory ribbon synapse. Cell. 2006:127(2):277–289. 10.1016/j.cell.2006.08.040. 17055430

[evae225-B87] Roychaudhuri R, Atashi H, Snyder SH. Serine Racemase mediates subventricular zone neurogenesis via fatty acid metabolism. Stem Cell Reports. 2023:18(7):1482–1499. 10.1016/j.stemcr.2023.05.015. 37352848 PMC10362503

[evae225-B88] Sambrook J, Fritsch EF, Maniatis T. Molecular cloning: a laboratory manual. 2nd ed. Cold Spring Harbor (NY): Cold Spring Harbor Laboratory Press; 1989.

[evae225-B89] Schlitter DA, Hutterer R, Maddalena T, Robbins LW. New karyotypes of shrews (Mammalia: Soricidae) from Cameroon and Somalia. Ann Carnegie Museum. 1999:68(1):1–14. 10.5962/p.226615.

[evae225-B90] Seidler NW . GAPDH and intermediary metabolism. Adv Exp Med Biol. 2013:985:37–59. 10.1007/978-94-007-4716-6_2. 22851446

[evae225-B91] Sharma V, Hecker N, Roscito JG, Foerster L, Langer BE, Hiller M. A genomics approach reveals insights into the importance of gene losses for mammalian adaptations. Nat Commun. 2018:9(1):1215. 10.1038/s41467-018-03667-1. 29572503 PMC5865188

[evae225-B92] Sharman GB . Chromosomes of the common shrew. Nature. 1956:177(4516):941–942. 10.1038/177941a0. 13322005

[evae225-B93] Shen Y-Y, Liang L, Li G-S, Murphy RW, Zhang Y-P. Parallel evolution of auditory genes for echolocation in bats and toothed whales. PLoS Genet. 2012:8(6):e1002788. 10.1371/journal.pgen.1002788. 22761589 PMC3386236

[evae225-B94] Sherman BT, Hao M, Qiu J, Jiao X, Baseler MW, Lane HC, Imamichi T, Chang W. DAVID: a web server for functional enrichment analysis and functional annotation of gene lists (2021 update). Nucleic Acids Res. 2022:50(W1):W216–W221. 10.1093/nar/gkac194. 35325185 PMC9252805

[evae225-B95] Simão FA, Waterhouse RM, Ioannidis P, Kriventseva EV, Zdobnov EM. BUSCO: assessing genome assembly and annotation completeness with single-copy orthologs. Bioinformatics. 2015:31(19):3210–3212. 10.1093/bioinformatics/btv351. 26059717

[evae225-B96] Smit A, Hubley R. RepeatModeler Open-1.0. 2023. [accessed 2023 January 7]. http://www.repeatmasker.org.

[evae225-B97] Smith MD, Wertheim JO, Weaver S, Murrell B, Scheffler K, Kosakovsky Pond SL. Less is more: an adaptive branch-site random effects model for efficient detection of episodic diversifying selection. Mol Biol Evol. 2015:32(5):1342–1353. 10.1093/molbev/msv022. 25697341 PMC4408413

[evae225-B98] Stanke M, Keller O, Gunduz I, Hayes A, Waack S, Morgenstern B. AUGUSTUS: ab initio prediction of alternative transcripts. Nucleic Acids Res. 2006:34(Web Server issue):W435–W439. 10.1093/nar/gkl200. 16845043 PMC1538822

[evae225-B99] Stankiewicz TR, Linseman DA. Rho family GTPases: key players in neuronal development, neuronal survival, and neurodegeneration. Front Cell Neurosci. 2014:8:314 10.3389/fncel.2014.00314. 25339865 PMC4187614

[evae225-B100] Stewart DT, Perry ND, Fumagalli L. The maritime shrew, *Sorex maritimensis* (Insectivora: Soricidae): a newly recognized Canadian endemic. Can J Zool. 2002:80(1):94–99. 10.1139/z01-207.

[evae225-B101] Suyama M, Torrents D, Bork P. PAL2NAL: robust conversion of protein sequence alignments into the corresponding codon alignments. Nucleic Acids Res. 2006:34(Web Server issue):W609–W612. 10.1093/nar/gkl315. 16845082 PMC1538804

[evae225-B102] Thomas WR, Dechmann DKN, Nieland J, Baldoni C, Carlson D, von Elverfeldt D, Holm-Jacobsen J, Muturi M, Corthals A, Dávalos LM. Molecular mechanisms of seasonal brain shrinkage and regrowth in *Sorex araneus*. bioRxiv 2023.10.02.560485. 10.1101/2023.10.02.560485., 3 October 2023, preprint: not peer reviewed.

[evae225-B103] Tollis M, Ferris E, Campbell MS, Harris VK, Rupp SM, Harrison TM, Kiso WK, Schmitt DL, Garner MM, Aktipis CA, et al Elephant genomes reveal accelerated evolution in mechanisms underlying disease defenses. Mol Biol Evol. 2021:38(9):3606–3620. 10.1093/molbev/msab127. 33944920 PMC8383897

[evae225-B104] Tomasi TE . Echolocation by the short-tailed shrew *Blarina brevicauda*. J Mammal. 1979:60(4):751–759. 10.2307/1380190. 750639

[evae225-B105] Uliano-Silva M, Ferreira JGRN, Krasheninnikova K, Darwin Tree of Life Consortium XYZ, Formenti G, Abueg L, Torrance J, Myers EW, Durbin R, Blaxter M, et al MitoHiFi: a python pipeline for mitochondrial genome assembly from PacBio high fidelity reads. BMC Bioinformatics. 2023:24(1):1–13. 10.1186/s12859-023-05385-y. 37464285 PMC10354987

[evae225-B106] Uniprot Consortium . UniProt: the universal protein knowledgebase in 2023. Nucleic Acids Res. 2023:51(D1):D523–D531. 10.1093/nar/gkac1052. 36408920 PMC9825514

[evae225-B107] Weisenfeld NI, Kumar V, Shah P, Church DM, Jaffe DB. Corrigendum: direct determination of diploid genome sequences. Genome Res. 2018:28(4):606.1. 10.1101/gr.235812.118. PMC5880249 29610250

[evae225-B108] White TA, Wójcik JM, Searle JB. Shrews, chromosomes and speciation. Shrews, chromosomes and speciation. Cambridge (UK): Cambridge University Press; 2019. p. 186–217.

[evae225-B109] Wójcik JM, Ratkiewicz M, Searle JB. Evolution of the common shrew, *Sorex araneus*: chromosomal and molecular aspects. Acta Theriol (Warsz). 2002:47(S1):139–167. 10.1007/BF03192485.

[evae225-B110] Wong JM, Folorunso OO, Barragan EV, Berciu C, Harvey TL, Coyle JT, Balu DT, Gray JA. Postsynaptic serine racemase regulates NMDA receptor function. J Neurosci. 2020:40(50):9564–9575. 10.1523/JNEUROSCI.1525-20.2020. 33158959 PMC7726548

[evae225-B111] Yamauchi T, Nio Y, Maki T, Kobayashi M, Takazawa T, Iwabu M, Okada-Iwabu M, Kawamoto S, Kubota N, Kubota T, et al Targeted disruption of AdipoR1 and AdipoR2 causes abrogation of adiponectin binding and metabolic actions. Nat Med. 2007:13(3):332–339. 10.1038/nm1557. 17268472

[evae225-B112] York NS, Sanchez-Arias JC, McAdam ACH, Rivera JE, Arbour LT, Swayne LA. Mechanisms underlying the role of ankyrin-B in cardiac and neurological health and disease. Front Cardiovasc Med. 2022:9:964675. 10.3389/fcvm.2022.964675. 35990955 PMC9386378

[evae225-B113] Zhang H, Ben Zablah Y, Zhang H, Jia Z. Rho signaling in synaptic plasticity, memory, and brain disorders. Front Cell Dev Biol. 2021:9:729076. 10.3389/fcell.2021.729076. 34671600 PMC8520953

[evae225-B114] Zhao J, Chen J, Li M, Chen M, Sun C. Multifaceted functions of CH25H and 25HC to modulate the lipid metabolism, immune responses, and broadly antiviral activities. Viruses. 2020:12(7):727. 10.3390/v12070727. 32640529 PMC7411728

