# Supplementary material for: Comparative Genomics of the World's Smallest Mammals Reveals Links to Echolocation, Metabolism, and Body Size Plasticity
Source: Genome Biol Evol. 2024 Oct 21;16(11):evae225. doi: 10.1093/gbe/evae225 (PMC11544316; doi:10.1093/gbe/evae225)
Supplement: evae225_Supplementary_Data [file evae225_supplementary_data.pdf]

Supplemental

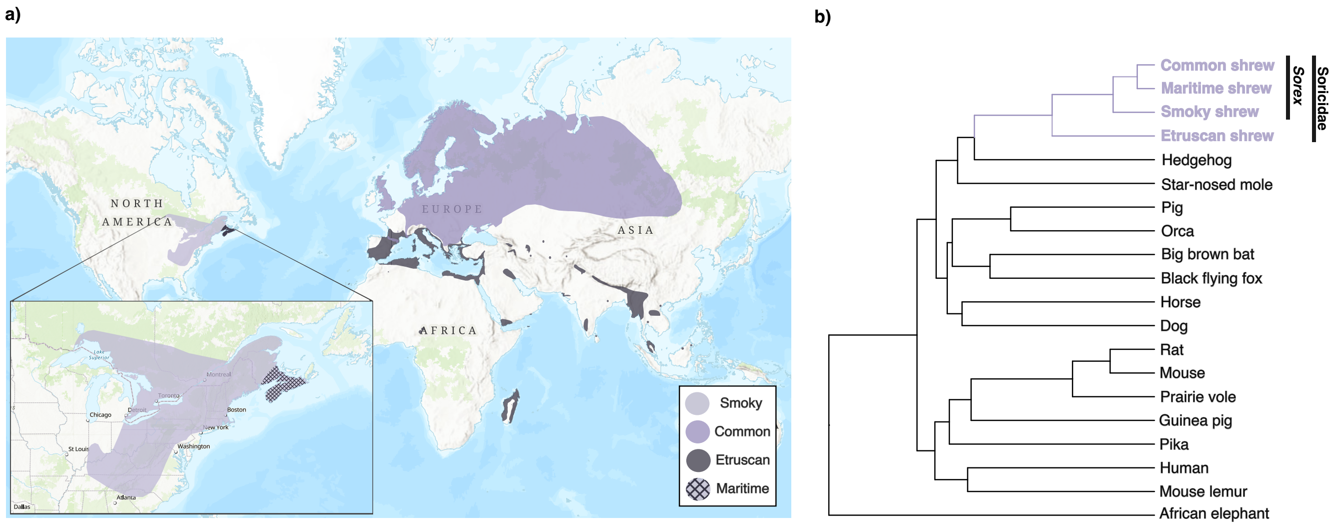

***Figure S1**. **a)** Shrew species' range according to the IUCN. **b)** Tree topology used for the accelerated region analysis.*

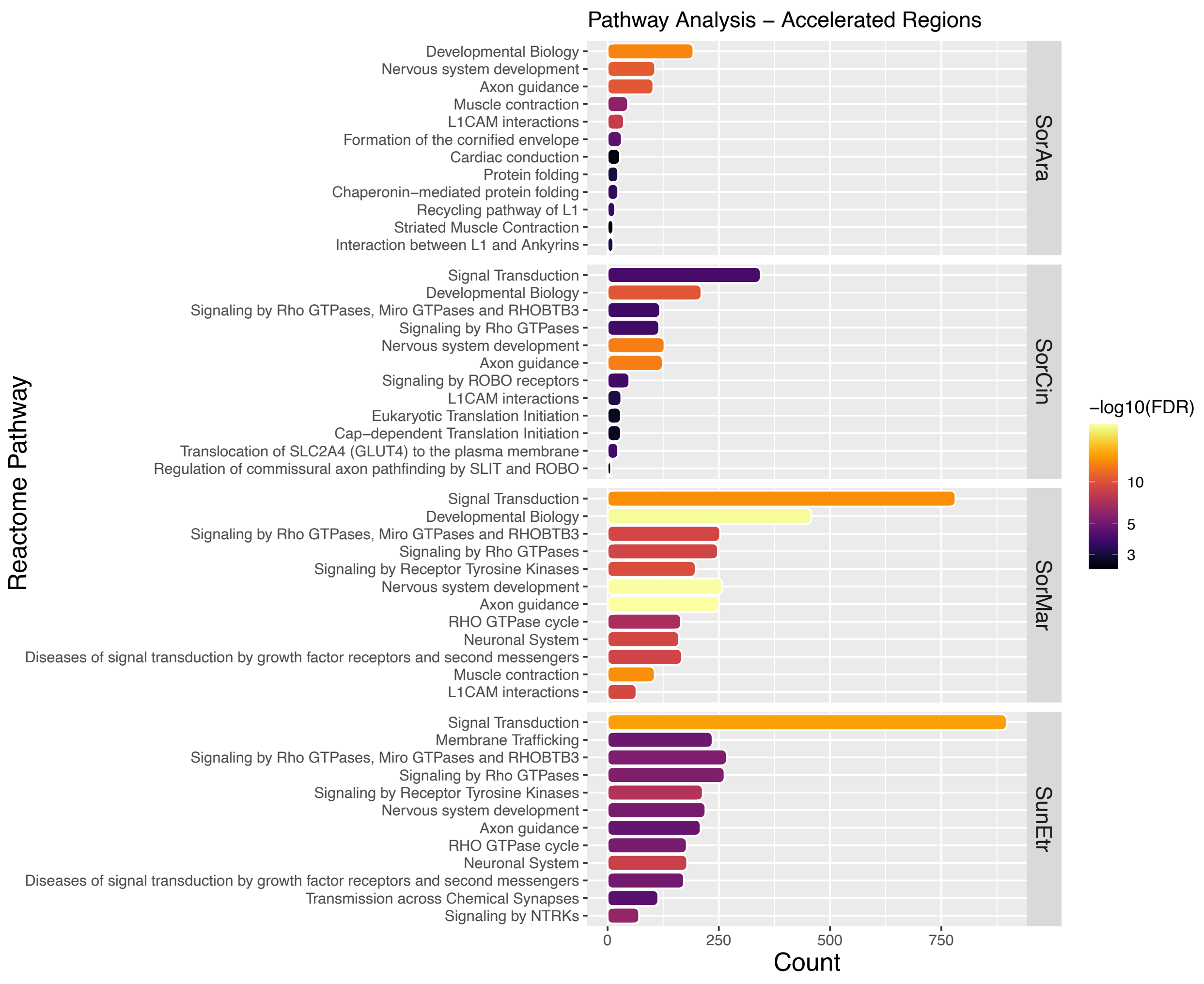

***Figure S2**. Top Reactome pathways for ARs found in each shrew species. All pathways are significant pathways (5% FDR).*

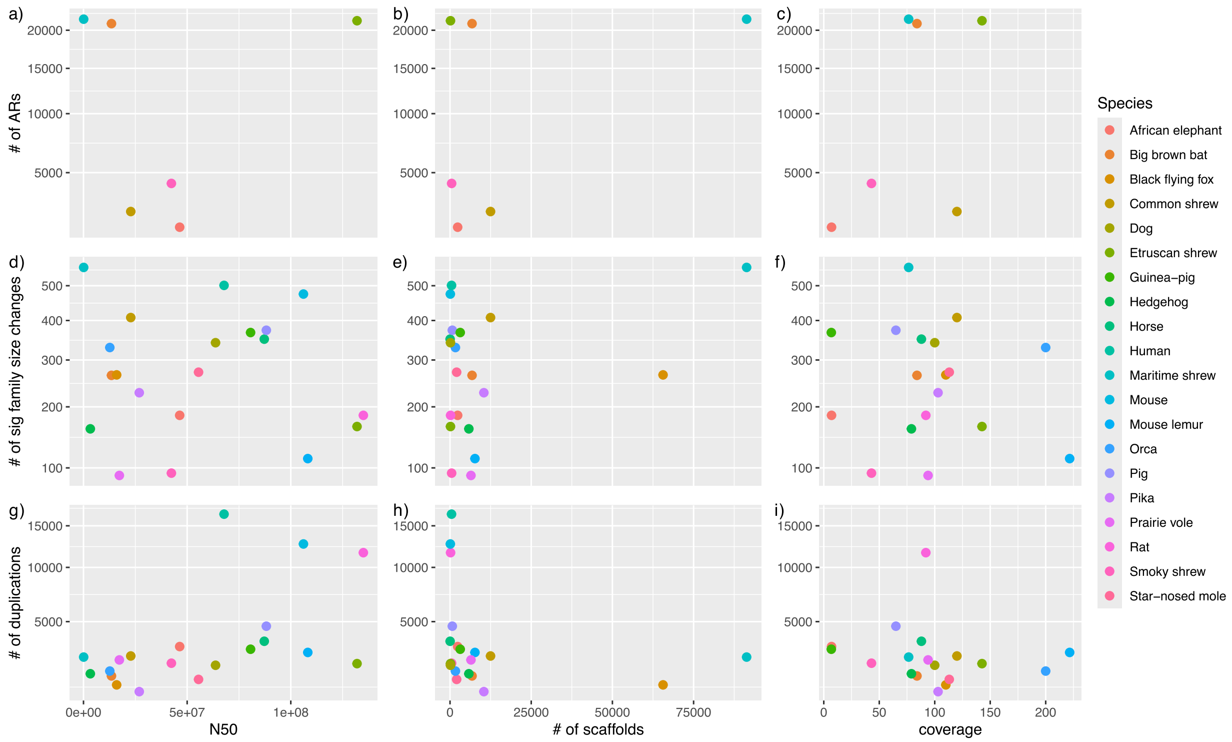

Figure S3*. Analysis results versus genome assembly statistics for each species. Number of ARs* ***a)*** *versus N50,* ***b)*** *number of scaffolds, and* ***c)*** *coverage. Number of gene families that underwent significant size change* ***d)*** *versus N50,* ***e)*** *number of scaffolds, and* ***f)*** *coverage. Number of gene duplications* ***g)*** *versus N50,* ***h)*** *number of scaffolds, and* ***i)*** *coverage.*

Table S1. *Conserved and accelerated region statistics aligned to the hg38 chromosomes (NCBI accession: GCA_000001405.15) for all shrew species, and the African elephant and big brown bat.*

**Chromosome**	**# 50 bp conserved regions**	**# ARs S. etruscus**	**# ARs S. fumeus**	**# ARs S. maritimensis**	**# ARs S. araneus**	**#ARs L. africana**	**#ARs E. fuscus**
**1**	53,160	1,494	548	2,183	259	67	2,870
**2**	62,618	2,638	315	1,262	145	362	1,942
**3**	45,064	1,131	365	1,317	139	177	1,253
**4**	28,793	962	156	615	104	73	1,266
**5**	38,505	983	219	1,393	212	38	1,475
**6**	32,985	1,358	325	871	144	93	422
**7**	31,500	988	124	939	124	158	915
**8**	26,899	986	233	512	67	86	1,166
**9**	29,383	778	154	735	73	178	436
**10**	26,248	2,080	153	841	126	77	928
**11**	30,470	1,056	244	1,667	260	65	1,144
**12**	27,793	1,237	344	1,371	197	28	175
**13**	18,667	536	121	395	39	190	324
**14**	24,584	805	80	806	73	10	343
**15**	20,174	1,143	104	816	111	54	1,146
**16**	21,988	651	187	1,138	117	70	1,890
**17**	26,095	978	166	1,600	181	15	228
**18**	16,292	355	77	348	30	54	1,685
**19**	13,331	457	142	1,575	126	41	768
**20**	13,448	421	120	609	70	35	51
**21**	4,008	103	19	120	14	24	467
**22**	6,175	214	76	468	32	9	45
**TOTAL**	**598,180**	**21,354**	**4,272**	**21,581**	**2,643**	**1,904**	**20,939**

Table S2. *Species name, genome version, and NCBI RefSeq accession number of the protein.faa and cds_from_genomic.fna files downloaded and used for the OrthoFinder and aBSREL analyses respectively. The synNet.maf files used for each species for the accelerated region analysis matched the genome versions listed here. *There is no RefSeq annotation file for the maritime shrew. We used the maritime shrew protein.faa file from our GenSAS annotation pipeline (available on gitlab).*

**Species**	**Genome version**	**NCBI RefSeq accession**
Black flying fox (*Pteropus alecto*)	ASM32557v1	GCF_000325575.1
Dog (*Canis lupus familiaris*)	CanFam6	GCF_000002285.5
Domestic guinea pig (*Cavia porcellus*)	Cavpor3.0	GCF_000151735.1
Star-nosed mole (*Condylura cristata*)	ConCri1.0	GCF_000260355.1
Big brown bat (*Eptesicus fuscus*)	EptFus1.0	GCF_000308155.1
Horse (*Equus caballus*)	EquCab3.0	GCF_002863925.1
Western European hedgehog (*Erinaceus europaeus*)	EriEur2.0	GCF_000296755.1
Human (*Homo sapiens*)	GRCh38.p14	GCF_000001405.40
African savanna elephant (*Loxodonta africana*)	Loxafr3.0	GCF_000001905.1
Gray mouse lemur (*Microcebus murinus*)	Mmur_3.0	GCF_000165445.2
Prairie vole (*Microtus ochrogaster*)	MicOch1.0	GCF_000317375.1
House mouse (*Mus musculus*)	GRCm39	GCF_000001635.27
Etruscan shrew (*Suncus etruscus*)	mSunEtr1.pri.cur	GCF_024139225.1
American pika (*Ochotona princeps*)	OchPri3.0	GCF_000292845.1
Killer whale (*Orcinus orca*)	Oorc_1.1	GCF_000331955.2
Norway rat (*Rattus norvegicus*)	mRatBN7.2	GCF_015227675.2
Common shrew (*Sorex araneus*)	SorAra2.0	GCF_000181275.1
Smoky shrew (*Sorex fumeus*)	SorFum_2.1	GCF_029834395.1
Maritime shrew (*Sorex maritimensis*)	SorMar_1.0*	GCA_030324115.1*
Pig (*Sus scrofa*)	Sscrofa11.1	GCF_000003025.6
